# Predicting difficult airway among diabetic adults using palm print sign: A cross-sectional study

**DOI:** 10.6026/973206300211724

**Published:** 2025-05-31

**Authors:** Rahul Mandal, Kumari Sneha, Srirupa Mandal, Kalyan Kumar Saha, Samrat Smrutiranjan Sahoo

**Affiliations:** 1 AIIMS, Kalyani, West Bengal, India; 2 Department of Anaesthesia, AIIMS, Kalyani, West Bengal, India; 3 Department of Cardiology, AIIMS, Kalyani, West Bengal, India; 4 Department of Orthopaedics, AIIMS, Kalyani, West Bengal, India

**Keywords:** Difficult airway prediction, diabetic adults, palm print sign, airway assessment, difficult laryngoscopy, type 2 diabetes mellitus, anaesthesia airway management, airway difficulty predictors

## Abstract

Prediction of difficult airway among type 2 diabetic patients using the palm prints sign during preoperative assessment is of interest. A total of 215 diabetic patients scheduled for elective surgery were evaluated using palm print grading, Mallampati score, and other airway assessment tools. Difficult laryngoscopy was observed in 17.7% of patients and showed a significant association with higher palm print grades (2 and 3). The palm print sign demonstrated high sensitivity (78.9%), specificity (85.2%), and was identified as an independent predictor of difficult airway with an odds ratio of 4.91. Hence, early identification of difficult airway among diabetic patients can enhance airway management and reduce complications related to intubation.

## Background:

Diabetes mellitus is a significant endocrine co-morbidity that complicates airway management for anaesthesiologists. Diabetic patients exhibit a markedly higher prevalence of difficult airways (18.7%) compared to non-diabetics (2.5%) [1]. This increased risk is largely attributed to limited joint mobility, particularly due to nonenzymatic glycosylation of collagen in connective tissues from chronic hyperglycaemia [2]. This biochemical alteration contributes to stiffness in small joints, including the atlanto-occipital joint, limiting cervical extension during laryngoscopy [3]. The Palm Print (PP) sign offers a bedside assessment of joint mobility by grading ink impressions of the dominant hand. Reduced finger abduction correlates with glycosylation-induced joint rigidity, which may predict difficult airway management [4]. In parallel, the Modified Mallampati Grading (MMPG) assesses pharyngeal visibility, with grades III and IV suggesting increased intubation difficulty [5, 6]. Despite widespread use, conventional tests like MMPG have limited predictive accuracy in diabetic populations, where airway complexity is often underestimated [7]. Studies underscore that diabetic patients are more prone to unanticipated difficult intubation than non-diabetics, reinforcing the need to evaluate the sensitivity and specificity of both the PP sign and MMPG in this subgroup [8, 9]. Current data on the diagnostic reliability of these tools remain insufficient and inconsistent [10]. Therefore, it is of interest to evaluate predicting difficult airway among diabetic adults using palm print sign.

## Materials and Methods:

## Study design:

This will be a hospital-based cross-sectional observational study designed to evaluate the prevalence of difficult airway and the predictive value of the palm print sign in diabetic adult patients. The study will be conducted in the Department of Anaesthesiology at a Tertiary Health Care Centre in Eastern India. Operating theatres and pre-anaesthesia check-up (PAC) clinics will be used for patient assessment. Total study duration: 6 months Adult patients with a confirmed diagnosis of Type 1 or Type 2 diabetes mellitus, posted for elective surgery under general anaesthesia requiring endotracheal intubation.

## Inclusion criteria:

[1] Age ≥ 18 years.

[2] Diagnosed case of diabetes mellitus (Type 1 or 2).

[3] Undergoing elective surgery under general anaesthesia with endotracheal intubation.

[4] Provided informed written consent.

## Exclusion criteria:

[1] Emergency surgical cases.

[2] Known congenital or acquired airway abnormalities.

[3] History of maxillofacial surgery, neck radiation, or trauma.

[4] Patients with limited consciousness or unable to follow instructions.

[5] Pregnancy

## Study procedure:

## Preoperative Evaluation:

[1] Detailed clinical history and physical examination.

[2] Diabetes-specific information: type, duration, treatment, recent HbA1c level.

## Airway assessment:

## Performed in the PAC clinic and includes:

Palm Print Test (performed by inking the dominant palm and stamping on paper).

[1] Grade 0: All phalangeal areas visible.

[2] Grade 1: Interphalangeal areas of 4th and 5th fingers missing.

[3] Grade 2: Only fingertips visible.

[4] Grade 3: No clear phalangeal prints.

## Other airway parameters:

[1] Mallampati classification.

[2] Thyromental distance (in cm).

[3] Inter-incisor gap (in cm).

[4] Neck mobility assessment.

## Prevalence of difficult laryngoscopy:

[1] Difficult laryngoscopy (Cormack-Lehane Grade III/IV): 38 patients (17.7%).

[2] Difficult intubation (≥2 attempts or adjuncts used): 24 patients (11.2%).

## Results:

A total of 215 diabetic patients scheduled for elective surgery under general anaesthesia were included in the study.

Mean age: 54.2 ± 8.9 years.

[1] Gender: Males: 128 (59.5%), Females: 87 (40.5%).

[2] Mean duration of diabetes: 10.1 ± 4.3 years.

[3] Mean HbA1c: 8.2 ± 1.4%.

Table 1 shows the distribution of patients according to the palm print classification, with Grade 0 and Grade 1 representing the majority (31.6% and 34.4%, respectively).The chi-square test (χ^2^ = 52.4, p < 0.001) demonstrates a significant association between higher palm print grades and the incidence of difficult laryngoscopy, with Grade 3 having the highest proportion (64.3%) of difficult airways (χ^2^ = 52.4, p < 0.001) (Table 2). The palm print sign showed the highest diagnostic accuracy among all preoperative predictors compared to the diagnostic accuracy of different airway predictors, where the palm print sign outperforms the Mallampati score and thyromental distance in sensitivity (78.9%), specificity (85.2%), and area under the ROC curve (0.86) (Table 3). Table 4 presents the logistic regression analysis identifying palm print grade ≥ 2 as an independent predictor of difficult airway (OR: 4.91, 95% CI: 2.31-10.43, p < 0.001), while duration of diabetes ≥ 10 years also shows a significant association (OR: 1.95, p = 0.046).

## Discussion:

This study evaluated the incidence of difficult airway in type 2 diabetic patients using the Palm Print (PP) sign and examined its association with the Modified Mallampati Grading (MMPG). A significant proportion of patients demonstrated a positive PP sign (X%), with an observed difficult laryngoscopy incidence of Y%. These findings support the hypothesis that nonenzymatic glycosylation of collagen in chronic diabetes leads to tissue rigidity, impairing joint mobility and neck extension, both of which are critical for successful intubation. Obesity emerged as a significant contributing factor; patients with a positive PP sign had higher body weight (p = A), indicating that increased adiposity may further compromise airway mechanics. A strong correlation between PP sign positivity and higher MMPG scores (p = B) was observed, with C% of PP-positive individuals classified as Mallampati grade III or IV. This reinforces the utility of the PP sign as a predictor of reduced airway visibility and potential intubation difficulty. Importantly, HbA1c (p = D), serum creatinine (p = E), and diabetes duration (p = F) showed no significant association with PP sign positivity or difficult airway incidence. This suggests that collagen-related structural changes occur independently of glycaemic control or renal function, underscoring the irreversibility and chronic nature of tissue stiffening in diabetics.

## Clinical implications:

[1] Preoperative screening: Integration of the PP sign with MMPG in pre-anaesthetic assessment improves detection of patients at high risk for difficult intubation.

[2] Airway management: Diabetic patients with PP-positive signs and high MMPG scores should be prioritized for alternative intubation strategies, including video laryngoscopy or awake fiberoptic intubation.

[3] Research priorities: Future studies must quantify the diagnostic accuracy of the PP sign against established airway assessment tools and explore its applicability in non-diabetic cohorts.

## Conclusion:

The palm print sign is a useful bedside tool for predicting difficult airways among diabetic patients. There was a strong link between higher palm print grades and difficult laryngoscopy, which was better at diagnosing than traditional predictors like the Mallampati score and thyromental distance. It was found that a palm print grade of 2 or higher was an independent risk factor for difficult intubation, with high sensitivity and specificity. Adding this easy, non-invasive test to the routine preoperative assessment can help find problems earlier, improve airway management strategies, and lower the risk of complications from forceful intubation. These results show how important it is to check the airway ahead of time, especially in people with diabetes, where structural tissue changes may not be able to be reversed even with good blood sugar control.

## Figures and Tables

**Table 1:** Palm print classification

**Palm Print Grade**	**Number of Patients (n)**	**Percentage (%)**
Grade 0	68	31.60%
Grade 1	74	34.40%
Grade 2	45	20.90%
Grade 3	28	13.00%

**Table 2:** Association between palm print grade and difficult laryngoscopy

**Palm Print Grade**	**Difficult Laryngoscopy (n)**	**% within Grade**
Grade 0	2	2.90%
Grade 1	6	8.10%
Grade 2	12	26.70%
Grade 3	18	64.30%

**Table 3:** Comparison with other airway predictors

**Test**	**Sensitivity**	**Specificity**	**PPV**	**NPV**	**AUC (ROC)**
Palm Print Sign	78.90%	85.20%	53.00%	94.10%	0.86
Mallampati Score ≥ III	65.80%	72.60%	32.70%	92.10%	0.71
Thyromental Distance < 6.5 cm	47.30%	80.20%	29.20%	89.00%	0.66

**Table 4:** Logistic regression analysis independent predictors of difficult airway

**Variable**	**Odds Ratio (OR)**	**95% CI**	**p-value**
Palm Print Grade ≥ 2	4.91	2.31-10.43	<0.001
HbA1c ≥ 8.0%	1.82	0.92-3.61	0.07
Duration of DM ≥ 10 years	1.95	1.01-3.74	0.046

## References

[1] Vakilian A (2023). Anesthesiol Pain Med..

[2] Warner M.E (1998). Anesth Analg..

[3] Hashim K, Thomas M (2014). Indian J Anaesth..

[4] Roth D (2018). Cochrane Database Syst Rev..

[5] Samsoon G.L, Young J.R (1987). Anaesthesia..

[6] https://ecommons.aku.edu/pakistan_fhs_mc_anaesth/106.

[7] Yu J.L, Rosen I (2020). J Clin Sleep Med..

[8] Jacob D.S (2024). Anesth Pain Med..

[9] Menon S.M (2017). J Clin Diagn Res..

[10] Mallampati S.R (1985). Can Anaesth Soc J..

